# Male fertility restored by transplanting primordial germ cells into testes: a new way towards efficient transgenesis in chicken

**DOI:** 10.1038/s41598-017-14475-w

**Published:** 2017-10-27

**Authors:** Pavel Trefil, Dorothea Aumann, Anna Koslová, Jitka Mucksová, Barbora Benešová, Jiří Kalina, Christine Wurmser, Ruedi Fries, Daniel Elleder, Benjamin Schusser, Jiří Hejnar

**Affiliations:** 1 BIOPHARM, Research Institute of Biopharmacy and Veterinary Drugs, 254 49 Jilove u Prahy, Czech Republic; 2 Reproductive Biotechnology, Technical University Munich, Liesel-Beckmann Street 1/III, 85354 Freising, Germany; 3 Institute of Molecular Genetics of the Czech Academy of Sciences, Videnska 1083, 14220 Prague, Czech Republic; 4 Chair of Animal Breeding, Technical University Munich, Liesel-Beckmann Street 1/III, 85354 Freising, Germany

## Abstract

The ongoing progress in primordial germ cell derivation and cultivation is opening new ways in reproductive biotechnology. This study tested whether functional sperm cells can be matured from genetically manipulated primordial germ cells after transplantation in adult testes and used to restore fertility. We show that spermatogenesis can be restored after mCherry-expressing or GFP-expressing primordial germ cells are transplantated into the testes of sterilized G_0_ roosters and that mCherry-positive or GFP-positive non-chimeric transgenic G_1_ offspring can be efficiently produced. Compared with the existing approaches to primordial germ cell replacement, this new technique eliminates the germ line chimerism of G_0_ roosters and is, therefore, faster, more efficient and requires fewer animals. Furthermore, this is the only animal model, where the fate of primordial germ cells in infertile recipients can be studied.

## Introduction

The germ line replacement technology has been driven by prospects of infertility treatment, genetic source conservation, and transgenesis. Particularly the transgenesis in chicken, still profoundly inefficient due to the difficulties in manipulating early embryos, relies on the transplantation of genetically modified primordial germ cells (PGC). Chicken became the first animal model, where the derivation, long-term *in vitro* culture, cryopreservation and reintroduction of PGC^1–3^ have been elaborated. This technology has opened a new approach to transgenesis in chicken, an alternative to the classic application of retrovirus vector into the subgerminal cavity of blastoderm embryos^4–6^.

Genetically modified PGCs have been shown to colonize, albeit with low efficiency, the germ line of chimeric roosters^1^. PGCs retain the capacity for homologous recombination, which enables gene targeting and the generation of immunoglobulin knockout chickens^7,8^. In spite of their success, the aforementioned approaches involve tricky techniques such as microinjection and the surrogate shell *ex vivo* culture system^9,10^. In one study^11^, the direct *in vivo* transfection of PGCs simplified the procedure, but the efficiency of germ line transmission in G_0_ chimeric roosters remained low.

Most technical obstacles posed by the avian reproduction system can be overcome by the reconstitution of gametogenesis from cultured and genetically manipulated precursors. While both spermatogenesis and oogenesis have been reconstituted in neonatal organ cultures in mice^12,13^, there has been limited progress in chickens. Spermatogenesis in irradiated roosters can be restored by the orthotopic transplanation of donor testicular cells^14,15^ and its efficiency increased by using GDNF family receptor α-1-positive spermatogonia precursors^16^. Even during a short-term culture, graft cells can be infected with retroviral vectors and transfected with gene reporters, resulting in a successful gene transfer^17,18^. The availability of PGC lines raised the question whether spermatogenesis could be restored by the orthotopic transplantation of these pluripotent cells. Previous studies have shown the low efficiency of germ line replacement after the transplantation of PGCs into embryonal gonads^19,20^. In this study, we present the first successful restoration of male fertility by the transplantation of reporter PGC lines into adult testes. This means that PGCs are able to accomplish the entire process of spermatogenesis, including meiosis and maturation in the microenvironment of spermatogenic epithelium. Since PGCs can be genetically manipulated *in vitro*, our approach offers innovative applications for transgenesis in chicken on the one hand and on the other hand the possibility to dissect spermatogenesis *in vivo*.

## Results

### Generation of mCherry-positive primordial germ cells

**Figure 1:**
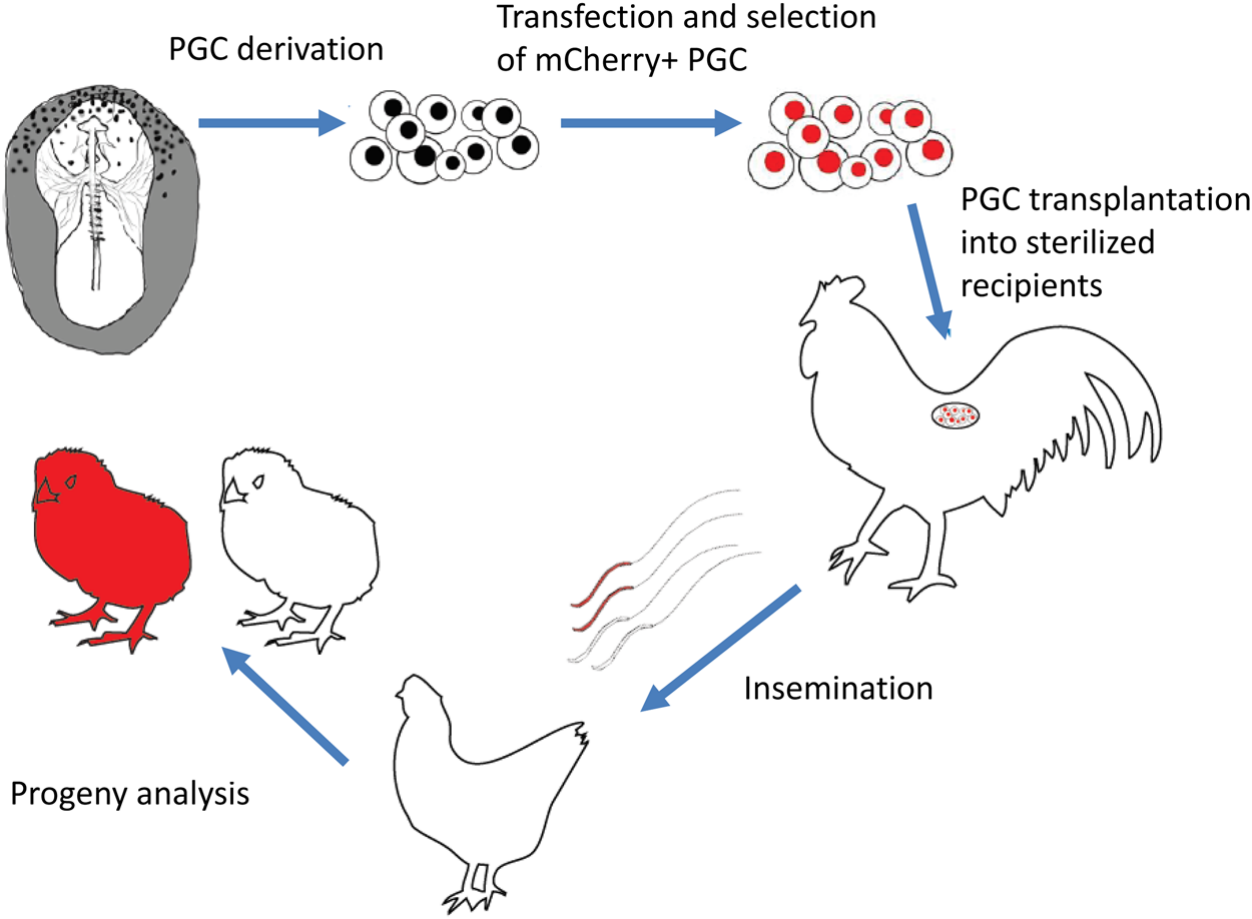
Schematic representation of PGC derivation, genetic modification, and transplantation into sterilized G_0_ roosters. This has been followed by insemination of hens and analysis of G_1_ offspring.

**Figure 2:**
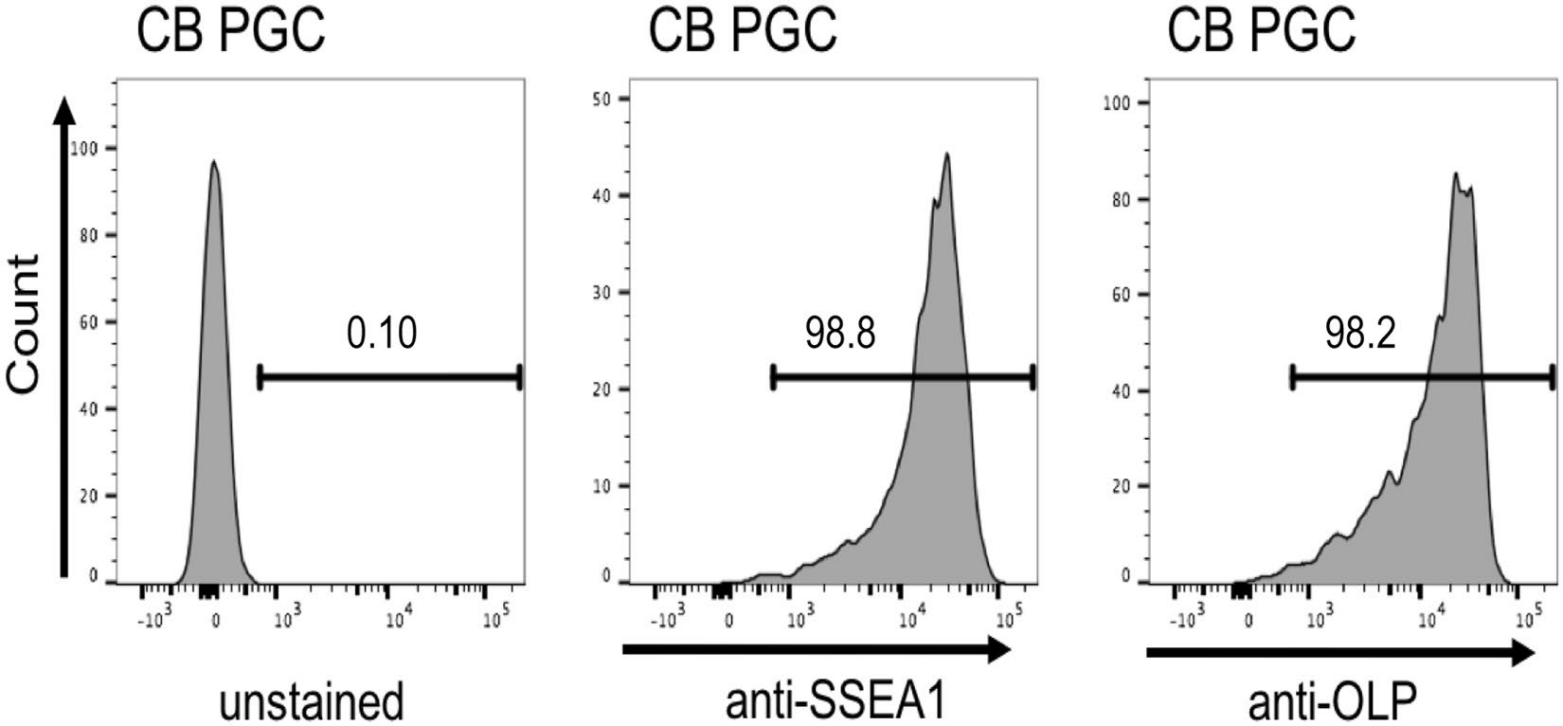
CB PGCs were derived from germinal crescent and stained for germline specific markers SSEA1 and chOLP. Expression was measured by flow cytometry.

**Figure 3:**
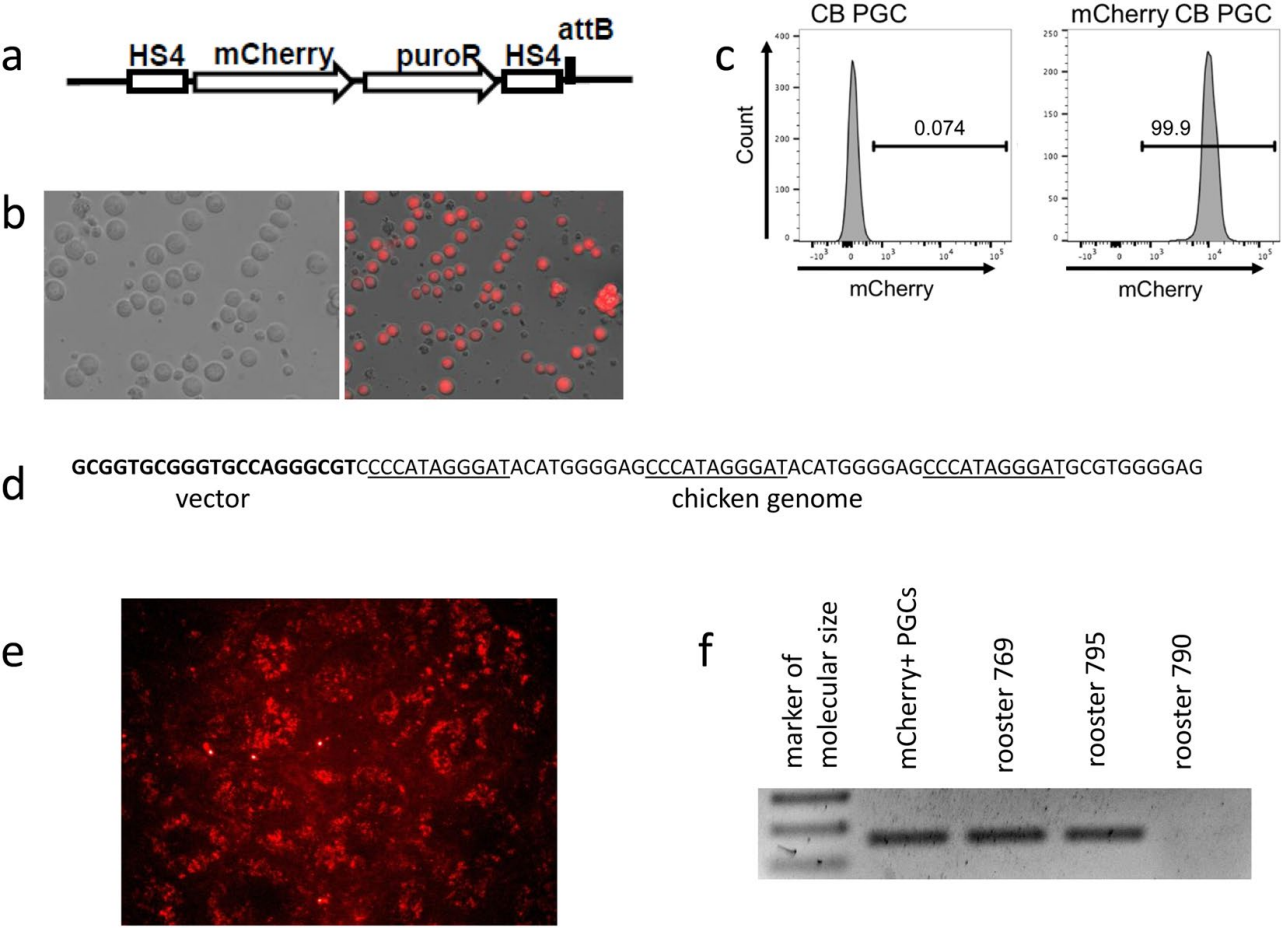
Analysis of modified PGCs and sperm of recipient rooster. (**a**) CB PGCs were transfected with a mCherry expression plasmid consisting of mCherry under a chicken β-actin promoter and a puromycin resistance gene under a CAG promoter flanked by HS4 insulators. (**b**) Succesful selection and expression of mCherry was confirmed by fluorescence microscopy and (**c**) flow cytometry. (**d**) The DNA junction between the mCherry vector and adjacent chicken genomic sequence detected in the whole-genome sequence of parental PGC clone. The vector-derived attB sequence is in bold and the repetitive motifs in the chicken genomic DNA are underlined. (**e**) mCherry positivity in the spermiogenic epithelium of G_0_ recipient rooster after orthotopic transplantation of mCherry-positive CB PGCs. (**f**) The mCherry reporter gene was detected by PCR in mCherry+ PGCs and semen samples of two recipient roosters.

The overall procedure (see Fig. 1 for a schematic) started with derivation of PGCs retaining the pluripotent capacity. PGCs were derived from the germinal crescent of a higly inbred CB chicken line^21^. Since this was the first time PGCs from such a highly inbred line were cultured, we used flow cytometry to test the cells for the SSEA-1 and OLP germline-specific markers. Over 98% of the cells tested positive (Fig. 2). The parental cell line was modified by inserting the mCherry gene under the control of the chicken β-actin promoter. To ensure the stable integration of non-concatemerized inserts, we used the phiC31 integrase-mediated integration^22^. After transfection of both insert (Fig. 3a) and integrase expression constructs, we selected four clonal cultures of mCherry-expressing CB PGCs out of the 5 × 10^6^ PGCs transfected (Fig. 3b,c). One of the four clones was characterized by whole-genome sequencing and the single mCherry integration was found in the chicken genomic DNA (Fig. 3d). Since the integration site resides in a highly repetitive region, its chromosomal position could not be determined. Preferential phiC31 integrase-mediated targeting of transgenes with attB sites into repetitive sequences was described previously^22^.

### Orthotopic transplantation of mCherry PGCs into sterilized roosters and restoration of spermatogenesis

The mCherry-positive CB PGCs were injected after 120 days in culture into both testes of four recipient G_0_ roosters (Table 1) sterilized previously by repeated irradiation. Three weeks after transplantation, one of the recipients was sacrificed and the presence of mCherry-positive cells in the testes was confirmed. mCherry-positive cells were found in all the layers of the seminiferous epithelium; they were also scattered throughout the interstitium (Fig. 3e). The restoration of spermatogenesis in the remaining three recipients was monitored for the next 12 months, the first spermatozoa being observed after 12 weeks. Spermatogenesis was restored in all three roosters. Importantly, semen samples from two recipients (numbers 795 and 769) tested mCherry-positive by PCR (Fig. 3f). These two roosters were used for further breeding to wild-type hens (Table 1). The titer of spermatozoa in their semen samples increased up to 10^4^–10^5^ per ml.

**Table 1:** Results of mCherry+ CB PGC transplantation and mCherry testing in G_1_ offspring.

Rooster No.	Spermatogenesis restored	mCherry+ semen	Number of hens inseminated	Number of fertilized eggs	Number of hatched chickens	mCherry+ chickens
769	+	+	2/12*	0/13*	10	6
790	+	—	—	—	—	—
795	+	+	2/21*	0/15*	14**	9
791	Sacrificed for section of testes					

*Intravaginal insemination/intramagnal insemination. **One of 14 hatched G_1_ chickens tested CB-negative.

Both standard intravaginal and intramagnal insemination of hens were used. However, only intramagnal insemination produced fertilized eggs, probably because of the low number of spermatozoa in the ejaculate. In the G_1_ progeny of both G_0_ roosters, mCherry-positive transgenic offspring were identified with frequencies of 60% and 64.3% (Table 1). The first mCherry +/− G_1_ hen hatched was dubbed Robin.

### Phenotypic analysis of G1 transgenic birds

**Figure 4:**
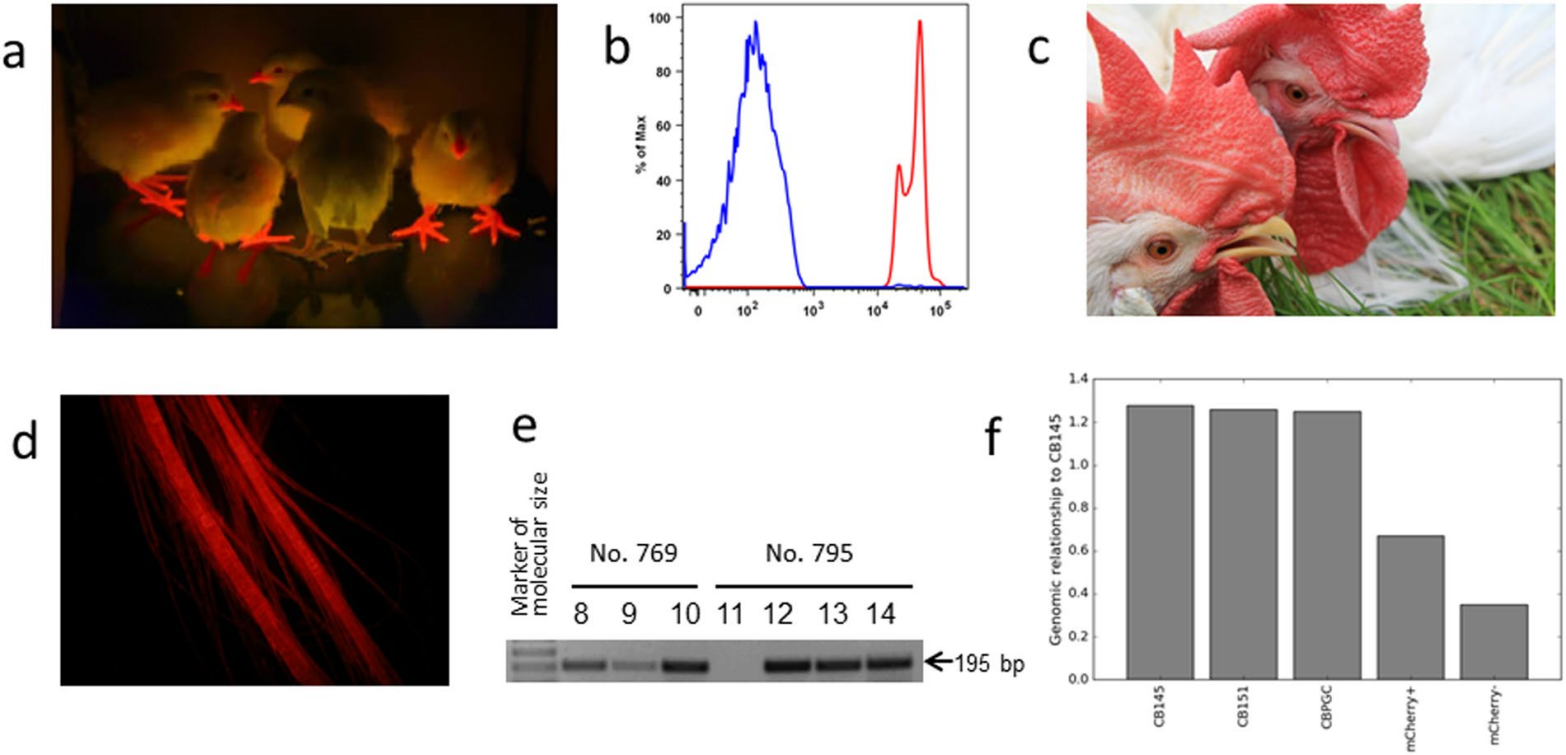
Phenotypic and genetic analysis of G_1_ offspring. (**a**) Freshly hatched G_1_ offspring, one mCherry-negative chicken in the middle. (**b**) Distribution of mCherry-positive cells in the peripheral blood. FACS histogram of mCherry signal in red blood cells (blue) and white blood cells (red). (**c**) mCherry positivity visible in the beak and featherless skin of the mCherry-positive rooster (right) in the daylight. The mCherry-negative wt rooster shown as a control (left). (**d**) Photostability of mCherry shown as mCherry signal in adult feather. (**e**) The 195 bp PCR product of the CB-specific tpn allele amplified in DNA of G_1_ offspring. The results of animals Nos 8 to 14 (from left to right) are shown, the non-CB chicken No. 11 is in the middle. Descendants of G_1_ roosters Nos 769 and 795 are indicated. (**f**) Genomic relationship of inbred individual CB145 with itself, inbred individual CB151, the primordial germ cell line, the mCherry+ chicken Robin and a mCherry- chicken. The genomic relationship was calculated based on about 1.6 million maximum quality SNP detected in 78 chickens.

**Figure 5:**
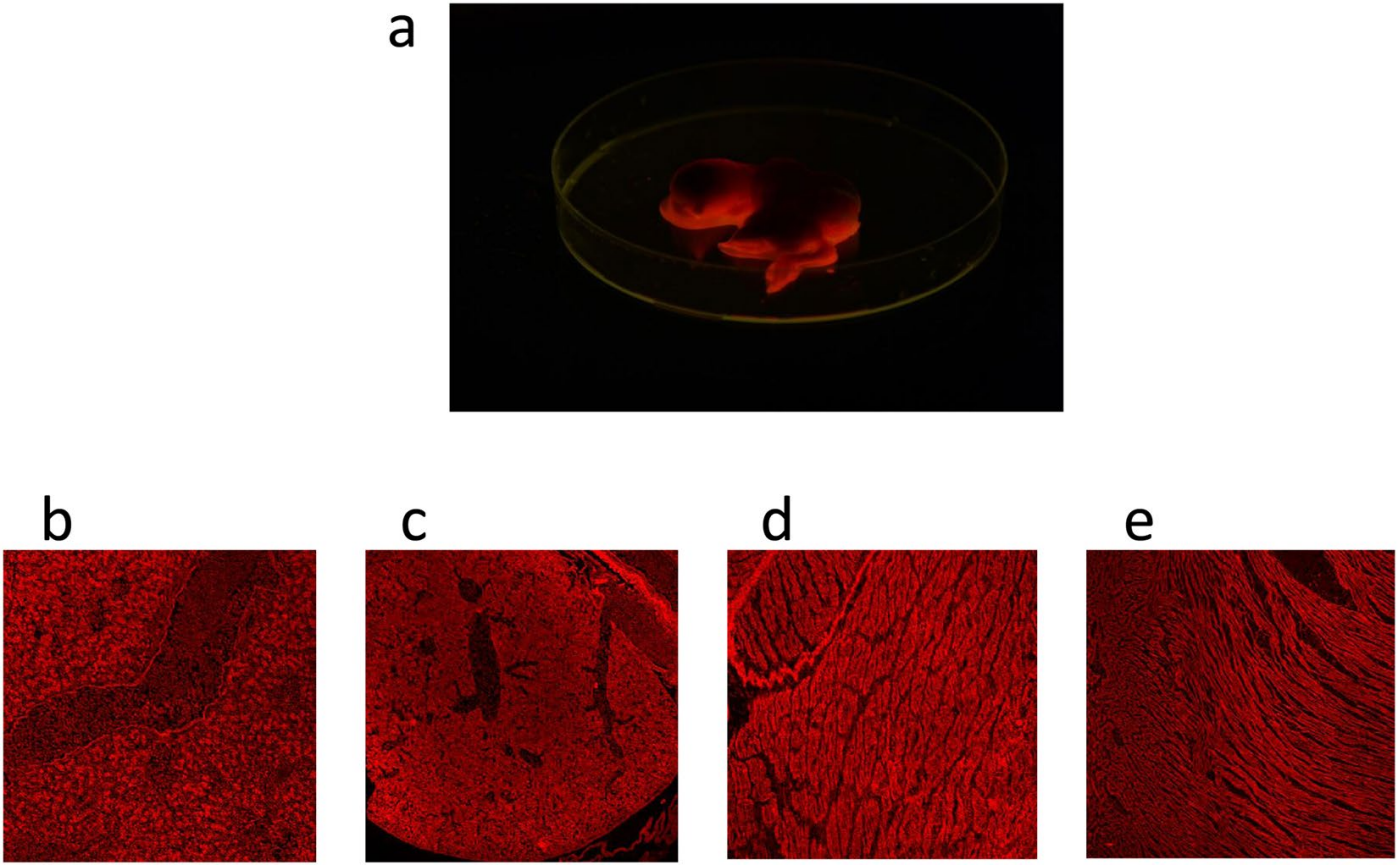
(**a**) mCherry-positive embryo in the middle of incubation. (**b**–**e**) mCherry positivity in embryo organs and tissues. From left to right: (**b**) liver, (**c**) spleen, (**d**) skeletal muscle, (**e**) heart.

The phenotype of mCherry-positive offspring was inspected by mCherry emission excited by the blue light of ca. 400–500 nm. When irradiated, the beaks, legs, and featherless parts of chicken body fluoresced red (Fig. 4a). mCherry positivity of organs and tissues was observed in embryos in the middle of incubation (Fig. 5a). Macroscopically, we observed mCherry positivity in all organs; this was confirmed by the fluorescence microscopy of organ sections, namely, liver, spleen, skeletal muscle, and heart (Fig. 5b–e). We used flow cytometry to test blood cells for mCherry positivity; in all the animals inspected, lymphoid cells were mCherry-positive (Fig. 4b). Interestingly, the expression of mCherry was visible even in daylight as a distinct reddish color of the beak, legs and featherless skin (Fig. 4c) of the G_1_ transgenic offspring. The enormous photostability of mCherry in adult birds is shown as mCherry positivity in feathers (Fig. 4d). In comparison to existing GFP chicken models, the detection of mCherry positivity is much easier and advantageous for *in vivo* imaging.

### Genetic analysis of G_1_ transgenic birds

To confirm the genetic succession of G_1_ progeny from the PGCs transplanted, we checked for the presence of the CB-specific allele of the transportin (*tpn*) locus in all the offspring. Out of the 21 G_1_ offspring, the CB-specific *tpn* allele was absent in just one mCherry-negative animal (Fig. 4e), which suggests that it arose from the residual spermatogenesis not completely abolished by the irradiation. Next, we compared the whole-genome CB sequence, the CB-derived mCherry PGCs, Robin, and the one mCherry-negative non-CB offspring (Fig. 4f). We confirmed the presence of the mCherry sequence at a single genomic site identical with the PGC line transplanted. The genomic relationship between the G_1_ offspring and CB line was analyzed based on the allele sharing of single-nucleotide polymorphisms (SNP) found in genome-wide sequences of 78 chickens. Two CB individuals were genetically identical with the mCherry PGC line (genomic relationships of 1.26 and 1.25), while the genomic relationship of Robin with the CB line amounted to 0.67 (Fig. 4f). This corresponds to the maternal contribution of an unrelated outbred hen to Robin’s genome. The genomic relationship of Robin’s mCherry-negative sibling lacking the CB-specific *tpn* allele with the CB line was 0.35, which points to its origin from the residual spermatogenesis of the G_0_ recipient rooster.

### Fertility of G_1_ offspring and mCherry transduction to G_2_

We crossed Robin the hen with mCherry-positive sibling in order to obtain the G_2_ generation. So far, we hatched 26 mCherry-positive and seven mCherry-negative G_2_ chickens, which fits with the expected ratio 3: 1. The phenotype of mCherry-positive G_2_ chickens was the same as in G_1_. Although we cannot yet quantitatively assess the fertility and viability of mCherry-positive birds, the production of G_2_ offspring indicates that mCherry-positive chickens are viable, fertile, and transduce the transcriptionally stable mCherry reporter to the next generation.

### Efficiency of transgenesis performed by mCherry PGCs insertion into embryonal circulation

To compare our transgenesis approach with the existing one as to efficiency, we injected mCherry-positive PGCs into H&H stage 14 embryos and raised the resulting male germline chimeras to sexual maturity. Their semen was then screened by semiquantitative PCR for the integration of the mCherry gene (data not shown), and the five most promising candidates were selected for breeding. We screened a total of 1167 embryos from five roosters. From rooster R011 we obtained one mCherry-positive embryo out of the 297 screened. This amounts to a germline transmission rate of 0.34% (Table 2). The integration of the mCherry gene was confirmed by mCherry-specific PCR on gDNA extracted from the positive embryo and a non-red sibling as negative control (data not shown).

**Table 2:** Results of mCherry transduction from different mCherry germline chimeras.

Chimeric rooster No.	Total number of tested embryos	mCherry-positive embryos
R009	245	0
R011	297	1 (0.34%)*
R021	338	0
R026	98	0
R035	189	0
Total	1167	1 (0.086%)*

*The efficiency of mCherry transduction in parenthesis.

### Independent orthotopic transplantation of eGFP+ PGCs confirms efficient transduction to G_1_

**Figure 6:**
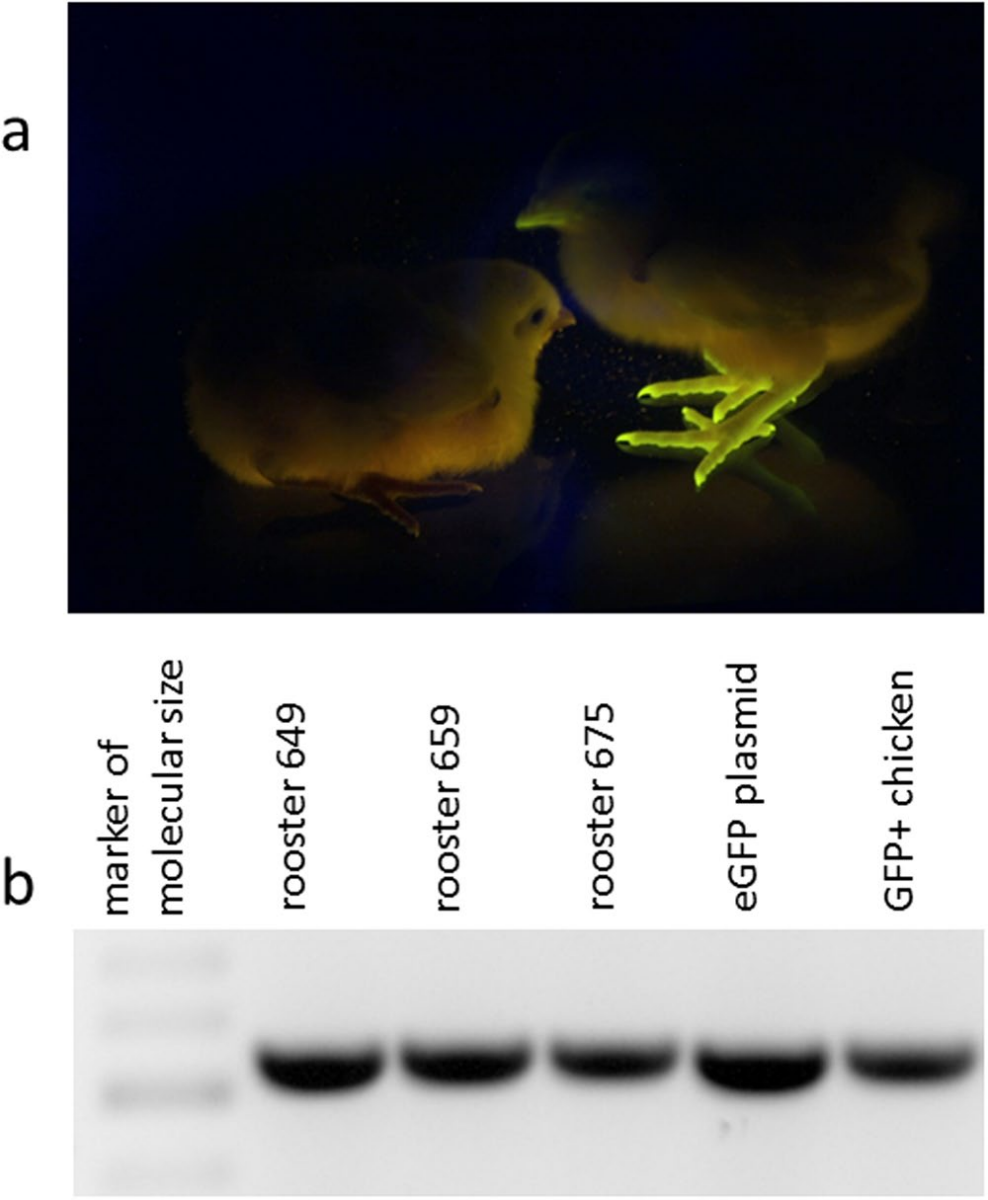
Phenotypic and genetic analysis of eGFP transduction. (**a**) Freshly hatched G_1_ offspring, one GFP-positive chicken right, wt chicken left. (**b**) PCR detection of eGFP transduction in the semen of roosters 649, 659, and 675 and in the GFP-positive G_1_ chicken No. 146. The eGFP-specific PCR fragment is 525 bp in length.

In order to confirm the high efficiency of fertility restoration and transgene transduction using the PGC transplantation into adult testes, we repeated the whole procedure from PGC manipulation to G_1_ analysis in an independent experiment. In this experiment, we used PGCs derived from commercial LSL breed harboring an eGFP selectable marker casette and transplanted higher amounts of them after 96 days of *in vitro* cultivation in both testes of five recipient roosters (Table 3). The procedure of recipient irradiation, orthotopic transplantation, and spermatogenesis monitoring were the same as in the first experiment with mCherry +PGCs. We sacrificed one of the recipients three weeks after transplantation and we detected GFP positivity in its testes (data not shown), The restoration of spermatogenesis in the remaining four recipients was monitored and we observed restoration of spermatogenesis in three roosters 12 weeks after transplantation. Semen samples from three recipients (numbers 649, 675, and 659) tested GFP-positive by PCR (Fig. 6b). Two roosters (Nos 659 and 675) were used for further breeding with wild-type hens by standard intravaginal insemination. We identified eGFP-positive transgenic offspring in the G_1_ progeny (Fig. 6) of both recipient roosters 675 and 659 with frequencies of 37.5% and 47% (Table 3).

**Table 3:** Results of eGFP+ PGC transplantation and GFP testing in G1 offspring.

Rooster No.	Spermiogenesis restored	GFP+ semen	Number of hatched chickens	GFP+ chickens
675	+	+	16	6
659	+	+	17	8
649	+	+	−*	−*
653	−	−	−	−
657	Sacrificed for section of testes			

*Recipient rooster No. 649 was used for further G_1_ breeding. Intravaginal insemination did not provide any offspring, intramagnal insemination was not performed.

## Discussion

In our study, we obtained viable genetically modified offspring from male PGCs matured in the adult testes of sterilized recipient roosters. These results highlight two fundamental novelties. First, chicken male PGCs are able to accomplish the entire process of spermatogenesis, including meiosis and maturation in the microenvironment of spermatogenic epithelium in the testes of adult recipients. Second, since PGCs can be genetically manipulated *in vitro*, our approach offers innovative applications for transgenesis in chicken. This capacity to maturate functional sperm from PGCs has so far been demonstrated just for embryonic testes^19,20,23^ where the efficiency varied from 0.6 to 40% in male PGCs transplanted into male recipients and from 0.1 to 0.3% in female PGCs transplanted into male recipients^19^. Similar efficiencies have been shown for PGC differentiation into functional oocytes in embryonic female recipients^19^. We conclude that the environment in testes retains, until adulthood, its permissiveness for sperm development from the PGCs. In one of two experiments described in this study, our approach required intramagnal insemination; anyway, its efficiency and reproducibility make it useful for transgenesis and germ line preservation. Based on the comparison of two experiments, we can also speculate that the restoration of spermatogenesis and the efficiency of artificial insemination might depend on the initial dose and culture time of transplanted PGCs. The epigenetic background of this PGC differentiation remains to be understood. It cannot be deduced from the mouse system, as chicken PGCs differ from their mammalian counterparts in histone marks of the epigenome^24^.

In comparison with the previously elaborated transplantation of spermatogonial cells into the testes of sterilized roosters^14^, PGCs provided similar results. Spermatogenesis was restored approximately four weeks earlier in the case of spermatogonial cells; however, PGCs proved more efficient in repopulating the seminiferous epithelium. The residual spermatogenesis in recipient roosters, which occured in previous experiments with spermatogonial cells, decreased the transplantation efficiency and distorted the ratio of expected phenotypes. However, our results with mCherry+ PGCs show that the residual spermatogenesis was very low (rooster No. 795) or nonexistent (rooster No. 769). The third transplanted rooster (No. 790) restored spermatogenesis, but mCherry-positivity was not detected (Table 1). Imperfect irradiation is the most probable explanation for this.

Unlike the existing methods used to produce germline chimeras and to screen for transgenic progeny in G_1_ generation, our procedure requires the repeated irradiation of recipients. However, we obtain transgenic progeny already in G_1_ generation - without any manipulation of early embryos, with much lower labor intensity, without any side effect of either recipients’ irradiation or surgery and with the use of much lower numbers of chickens. This is in line with the 3R concept (refinement-reduction-replacement) in animal technology and testing.

Importantly, we have done one of our experiments with PGCs derived from inbred CB embryos. In the future, we plan to inseminate CB hens and obtain the mCherry transgene on the inbred CB background. The availability of inbred transgenic animals makes it possible for adoptive immune transfer, cell lineage transfer, and cell lineage tracking to be used as tools in developmental biology and immunology. Furthermore, the mCherry-positive chicken adds another fluorescent marker to the existing GFP system. Last but not least, we created a model, which makes possible to learn more about factors important in the development of germ cells *in vivo*.

## Methods

### Animals

Inbred chicken line CB used for PGC derivation was maintained at the Institute of Molecular Genetics, Prague^21^. Embryos of commercial chicken breed LSL used for PGC derivation were obtained from Lohmann Tierzucht GmbH (Cuxhaven, Germany). Seven-month-old outbred White Leghorn (WL, line CC21I × L15) roosters were used as recipients of PGCs. Outbred Barred Leghorn hens (line SH) were used in insemination experiments in this study. Hens were housed individually in battery cages, roosters were maintained in deep litter individual cages under standard husbandry conditions with food and water provided ad libitum and 12 h light: 12 h dark cycle. Eggs were incubated in a forced air incubator (BIOS MIDI, Czech Republic) adjusted for fowl egg incubation conditions. All birds were obtained from the Institute of Molecular Genetics, Prague. We performed all experiments in accordance with Czech legislation for animal handling and welfare. Chickens for the injections of PGCs and germline testing were kept at the Institute for Animal Physiology, LMU Munich. Animal experiments were approved by the Regierung von Oberbayern (AZ: 55.2-1-54-2532-104-2015) and by the Animal Commodities Department of the Ministry of Agriculture of the Czech Republic (3980/2015-MZE-17214).

### Derivation and culture of parental PGCs

Fertilized eggs from the CB chicken line or LSL parental stocks were incubated for 35 hours. The germinal crescent was dissected and PGCs were isolated as described before^25^. PGCs were cultured in Avian KO-DMEM supplemented with growth factors as described elsewhere^26^.

### Generation and transfection of reporter PGC lines

An eGFP plasmid that was used before to generate transgenic chickens^25^ was used to swap the eGFP with the mCherry gene by Gibson Assembly (New England Biolabs) using the following oligos: fw: 5′-TATCGCATGCCTGCGATGGTGAGCAAGGGCGAGG-3′, rev: 5′-GCATGGACGAGCTGTACAAGTAGAACTTGTTTATTGCAGCT-3′. Correct sequence was confirmed by sequencing. To generate mCherry expressing PGCs, 5 × 10^6^ CB PGCs were resuspended in Nucleofector Solution V (Lonza) and electroporated using 10 µg of mCherry plasmid and 10 µg phi31C Integrase in a total volume of 100 µl. Electroporation was carried out using a ECM 830 Square Wave Electroporation System (BTX) using eight square wave pulses (350 V, 100 µsec). After transfection, cells were plated on 48-well plates. Selection with puromycin (0.5 µg/ml) was started after 3–5 days. To generate eGFP positive PGCs, a construct harbouring a chicken beta-actin promotor controlling eGFP expression and a puromycin resistence gene driven by the CAG promoter was used. LSL PGCs were transfected and selected as described for the mCherry construct.The presence of mCherry and eGFP genes in PGCs and later in recipient rooster sperms and G_1_ offspring was detected by following primers: 5′-CAGGACGGCGAGTTCATCTACAAG-3′ and 5′-CAGCTTCAGCCTCTGCTTGATCTC-3′ (for mCherry) and 5′ TGACCCTGAAGTTCATCTGCA 3′ and 5′-ACGAACTCCAGCAGGACCATGT-3′ (for eGFP). We amplified specific fragments in 35 cycles of denaturation at 95 °C for 30 sec, annealing at 55 °C for 30 sec. and elongation at 72 °C for 50 sec.

### FACS

CB PGCs were stained for the germline specific markers SSEA1 and ovomucin-like-protein (OLP). Anti-murine-SSEA-1 antibody (eBioscience) was used in a dilution of 1:10000. Unpurified anti-chicken OLP-supernatant (Willi Halfter, University of Pittsburgh) was used at a dilution of 1:500. Primary antibodies were detected with a goat-anti-mouse-IgM-APC (Jackson ImmunoResearch). CB PGC FACS analyses were performed using a BD FACS Canto II (BD Bioscience).

For the FACS analysis of mCherry positivity in blood cells, the heparinized blood samples were centrifuged in the 1.077 density Histopaque gradient (Sigma-Aldrich) to separate nucleated erythrocytes and peripheral blood mononuclear cells (PBMC). Cells were analyzed using a FacsCalibur device (Becton Dickinson).

### Irradiation treatment of recipient roosters

The testes of all G_0_ recipient roosters used in this experiment were irradiated to destroy their endogenous spermatogenesis. A Theragam K-01 radiation unit (Škoda UJT Zbraslav, Czech Republic) using ^60^Co as a source of gamma rays was used for the irradiation procedure as described previously^27^. Each rooster was subjected to a series of 5 doses of 8 Gy over 14 days. Two weeks after the last irradiation the first attempts to semen collection started. When semen samples were evaluated as azoospermic in 4 consecutive observations, roosters were considered ready for transplantation. None of the sterilized roosters died due to the irradiation or showed any side effects related to the radiation procedure.

### Orthotopic transplantation of PGCs into the testes of irradiated roosters

Recipient roosters were anesthetised with intramuscular injection of 15 mg/kg ketamine, (Narkamon, Bioveta, Czech Republic) and 4 mg/kg xylazine (Rometar, Bioveta, Czech Republic). The total dose of 5 × 10^5^ mCherry+ or 5 × 10^6^ of GFP+ PGC was applied in 250 μl of cell suspension into each testis by injection through tunica albuginea at four to five different randomly distributed locations. The transplantation procedure did not cause any side effects or mortality. The mCherry+ PGCs were applied after 120 days in culture, the GFP+ PGCs were cultured for 96 days.

### Sperm collection and insemination

The attempts of semen collection from PGC-transplanted recipients started 3 weeks after transplantation. Semen samples were collected using the dorso-abdominal massage. Intramagnal insemination was performed to increase the probability of fertilisation. The insemination dose ranged from 0.1–0.4 ml of fresh undiluted semen per hen with semen concentration 10^4^–10^5^ sperms/ml. The hens were inseminated intravaginally (the eGFP experiment) or intramagnally (mCherry experiment). For intramagnal insemination, the hens were anesthetised with intramuscular injection of Narkamon and Rometar (see above the dosage). Fresh ejaculate was injected directly into the magnum within 10 min after collection.

### Geno- and phenotyping of progeny

The presence of CB DNA was detected by amplifying the CB-specific tpn locus in the MHC region. Forward and reverse primers sTPN-CB (5′-TCATCCAGTGCTCACCCCATG-3′) and aTPN-CB (5′-GACCACGAGGAGCAACGAAGG-3′) were used for 30 cycles of 95 °C 15 sec, 62 °C 30 sec and 72 °C 20 sec. The PCR amplifies a 195 bp product exclusively of CB DNA^28^.

Paired-end libraries were prepared from DNA samples of the of inbred chickens CB145 and CB151, the CB-derived primordial germ cell line, the mCherry+, and the mCherry- individual using the TrueSeq kit (Illumina) and sequenced on a HiSeq. 2500 instrument. The resulting 125-basepair reads were aligned to the reference sequence of the chicken genome (Gallus_gallus_4.0) using the BWA-MEM algorithm^29^. Individual files in SAM format were converted into BAM format using SAMtools^30^. Duplicate reads were marked with the MarkDuplicates command of Picard Tools (http://broadinstitute.github.io/picard/). Single nucleotide polymorphisms (SNPs) and short insertion and deletion polymorphisms (INDELs) were genotyped in totally 78 individuals with an average genome coverage of 14.25 using the multi-sample approach implemented in mpileup of SAMtools along with BCFtools^30^. Genomic reliationships were estimated using the make-rel method of PLINK 1.9^31^ based on 1,637,853 unlinked SNPs with a quality value of at least 998.

Both mCherry and GFP positivity *in vivo* was tested in blue light of 400–500 nm using the Dark Reader Transilluminator (Clare Chemical Research, Dolores, CO, USA).

### Generation of germline chimeras and germline testing

In order to test the capability of mCherry CB PGCs to colonize the gonads and go germline after injection into H&H 14 embryos we injected 3000 cells per embryo and raised the resulting male embryos to sexual maturity like described before^7^. Semen was collected by abdominal massage and isolated gDNA was tested with primers specific for mCherry. For detection of mCherry gene, the following primers were used: 5′-CAGGACGGCGAGTTCATCTACAAG-3′ and 5′-CAGCTTCAGCCTCTGCTTGATCTC-3′. 5x FIREPol MasterMix (Solis BioDyne) was used for all PCRs according to manufacturer’s protocol. Roosters with mCherry positive semen were bred to white leghorn hens and the resulting eggs were icubated at 37°C. On embryonic day seven, several eggs were cracked and mCherry positive embryos were visualized by using a Leica stereomicroscope.
